# Colpocephaly Diagnosed in a Neurologically Normal Adult in the Emergency Department

**DOI:** 10.5811/cpcem.2019.9.44646

**Published:** 2019-10-21

**Authors:** Christopher Parker, Wesley Eilbert, Timothy Meehan, Christopher Colbert

**Affiliations:** University of Illinois College of Medicine, Department of Emergency Medicine, Chicago, Illinois

## Abstract

Colpocephaly is a form of congenital ventriculomegaly characterized by enlarged occipital horns of the lateral ventricles with associated neurologic abnormalities. The diagnosis of colpocephaly is typically made in infancy. Its diagnosis in adulthood without associated clinical symptoms is exceptionally rare. We report a case of colpocephaly diagnosed incidentally in an adult without neurologic abnormalities in the emergency department. To our knowledge, this is only the ninth reported case in an asymptomatic adult and the first to be described in the emergency medicine literature.

## INTRODUCTION

Colpocephaly is a rare form of congenital ventriculomegaly often associated with partial or complete agenesis of the corpus callosum. Diagnosis is typically made in infancy due to associated neurological and neurodevelopmental disorders.^1^,^2^ Initial discovery in adulthood is exceedingly rare.^3^–^9^ When identified incidentally in adults, colpocephaly may be misdiagnosed as hydrocephalus.^4^,^7^,^9^ We report a case of colpocephaly in an adult of normal neurological development discovered in the emergency department (ED).

## CASE REPORT

A 29-year-old male with no pertinent past medical history presented to our ED with two weeks of intermittent headaches. His headache was described as throbbing in character, localized to the bitemporal region, non-radiating, and non-positional. The headache occurred daily, lasting a few minutes to hours, with no particular exacerbating factors. His symptoms improved with acetaminophen, which he used sparingly. He reported no associated vomiting, gait abnormalities, vision changes, confusion, urinary changes, or other neurologic abnormalities. He had been treated at four different EDs in the two weeks prior to presentation for the headaches, but no imaging studies had been performed. The patient had no psychiatric history. His highest level of education was a high school diploma, and he was unemployed.

On arrival, the patient was afebrile with pulse, blood pressure, and respiratory rate all within the normal range. Physical examination revealed an anxious male who was alert, oriented, and in no acute distress. His head was normocephalic with no evidence of trauma. His pupils were equal, round, and reactive to light. His neurological examination did not reveal any cranial nerve deficits, speech abnormalities, muscle weakness, or loss of sensation. His reflexes were intact and symmetrical. His coordination was normal. His gait was stable with balanced cadence, and he exhibited a negative Romberg test. His visual acuity was 20/20 in both eyes. The remainder of the examination was unremarkable.

**Image 1:**
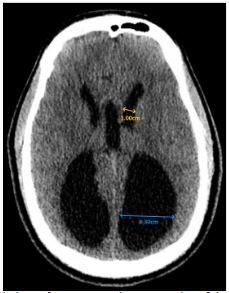
Axial view of a computed tomography of the head demonstrating ventriculomegaly, consistent with colpocephaly. Lines compare the maximal width of the occipital horns (blue line) to that of the anterior horns (yellow line) of the lateral ventricle, with an occipital-to-anterior horn ratio of 4.3.

**Image 2:**
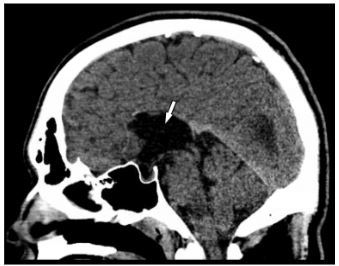
Sagittal view of computed tomography of the head demonstrating complete agenesis of the corpus callosum (arrow).

Laboratory values of complete blood count and complete metabolic panel were unremarkable. The serum carboxyhemoglobin level was within the normal range. Due to the patient’s headache not being fully consistent with a primary headache and his multiple visits to the ED without a history of imaging, computed tomography (CT) of the head was ordered to assess for a possible anatomic cause of his symptoms. The CT was notable for marked enlargement of the occipital horns of the lateral ventricles with agenesis of the corpus callosum, consistent with colpocephaly (Images 1 and 2).

The patient was evaluated by the neurology service in the ED. It was their opinion that his headaches were primary in nature and not associated with the incidental finding of colpocephaly. His headache resolved after receiving 10 milligrams (mg) of intravenous metoclopramide and 50 mg of oral diphenhydramine, and he was discharged home with neurology follow-up. The patient returned to the ED one month later for an unrelated complaint and did not report a headache at that time.

**CPC-EM Capsule**

What do we already know about this clinical entity?

*Colpocephaly is a congenital form of ventriculomegaly. Diagnosis is typically made in infancy due to associated neurologic abnormalities*.

What makes this presentation of disease reportable?

*This is only the ninth reported case of colpocephaly diagnosed in an asymtomatic adult and the first to be described in the emergency medicine literature*.

What is the major learning point?

*While exceptionally rare, colpocephaly may be present in asymptomatic adults. It may be misdiagnosed in adults as normal pressure hydrocephalus*.

How might this improve emergency medicine practice?

*Knowledge of the clinical and radiographic differences between colpocephaly and normal pressure hydrocephalus will help avoid unnecessary diagnostic and therapeutic procedures*.

## DISCUSSION

First described by Benda in 1940, colpocephaly is a rare congenital brain malformation in which the occipital horns are disproportionately larger than the anterior horns of the lateral ventricles.^10^ Colpocephaly can be associated with partial or complete agenesis of the corpus callosum, Chiari malformations, lissencephaly, and microcephaly.^9^ The abnormal ventricular enlargement in colpocephaly is believed to be secondary to the developmental arrest of white matter formation that occurs during fetal development.^7^ Various etiologies have been proposed, including chromosomal abnormalities, intrauterine infection, perinatal anoxic-ischemic encephalopathy, intrauterine growth retardation, and maternal toxin exposure.^1^,^3^

Colpocephaly is typically discovered in infancy due to associated intellectual disability, seizures, motor abnormalities, or visual abnormalities.^1^,^2^ Discovery in adulthood is remarkably uncommon and has only been reported eight times previously (Table 1).^3^–^9^ Colpocephaly can be identified radiographically by measuring the maximal width of the anterior and occipital horns of the lateral ventricles. An occipital-to-anterior horn ratio of greater than 3 is highly specific for colpocephaly, although it has relatively low sensitivity.^3^,^11^

The identification of colpocephaly in adulthood is a phenomenon that has only recently been described. Colpocephaly discovered in adulthood may be misdiagnosed as normal pressure hydrocephalus, a much more common cause of ventriculomegaly in adults.^3^,^5^ Knowledge of the clinical and radiographic differences between these two conditions is needed to avoid unnecessary diagnostic and therapeutic procedures (Table 2). Colpocephaly discovered incidentally in asymptomatic adults requires no specific treatment.

## CONCLUSION

Colpocephaly discovered in asymptomatic adults is exceedingly rare. It may be misdiagnosed as normal pressure hydrocephalus in the ED. It is important to differentiate between these two conditions to avoid unnecessary interventions.

## Figures and Tables

**Table 1:** Previous reported cases of colpocephaly diagnosed incidentally in adulthood.

Author	Year	Patient	Reason for imaging
Wunderlich G, et al.^6^	1996	60-year-old female	New onset partial complex seizures
Cheong J, et al.^7^	2012	67-year-old female	Four months of headache and dizziness - ultimately diagnosed with meningioma
Esenwa C, et al.^3^	2013	60-year-old female	Headache after minor head trauma
Ciurea R, et al.^9^	2014	28-year-old female	Longstanding, intermittent headaches and vertigo
Brescian N, et al.^8^	2014	88-year-old male	New onset left hand apraxia
Nasrat T, et al.^4^	2014	66-year-old female	One month of declining mental status - ultimately diagnosed with paraspinal abscess
Bartolome E, et al.^5^	2016	67-year-old female	Syncopal episode
		60-year-old female	Confusion with fever - ultimately diagnosed with an upper respiratory infection

**Table 2:** Clinical and radiographic characteristics of colpocephaly and normal pressure hydrocephalus.

	Colpocephaly	Normal pressure hydrocephaly^3^,^12^
Clinical characteristics	Typically diagnosed in infancy due to associated neurological abnormalities	Typically diagnosed after age 50 years
	Diagnosis in asymptomatic adults is exceptionally rare	Symptoms include varying degrees of the classic triad of gait disturbance, urinary incontinence, and dementia
Radiographic characteristics	Disproportionate dilation of the occipital horns of the lateral ventricles, often associated with full or partial agenesis of the corpus callosum	Ventriculomegaly marked by dilation of the anterior and occipital horns of the lateral ventricles
Treatment	No treatment is indicated when diagnosed in asymptomatic adults	CSF shunting procedures lead to symptom improvement in approximately 60% of cases

*CSF*, cerebral spinal fluid.
